# *Mycoplasma bovis* antibody dynamics in naturally exposed dairy calves according to two diagnostic tests

**DOI:** 10.1186/s12917-018-1574-1

**Published:** 2018-08-30

**Authors:** Mette Bisgaard Petersen, Nadeeka K. Wawegama, Matthew Denwood, Philip F. Markham, Glenn F. Browning, Liza Rosenbaum Nielsen

**Affiliations:** 1 Department of Veterinary and Animal Sciences, Faculty of Health and Medical Sciences, University of Copenhagen, Grønnegårdsvej 8, 1870 Frederiksberg, Denmark; 2 Asia-Pacific Centre for Animal Health, Melbourne Veterinary School, Faculty of Veterinary and Agricultural Sciences, The University of Melbourne, Parkville, VIC 3010 Australia

**Keywords:** *Mycoplasma bovis*, ELISA, BioX bio K 302, MilA ELISA, Dairy calves, Antibody

## Abstract

**Background:**

Inexpensive and convenient diagnostic tests for use in clinical work and for the surveillance of infection with *Mycoplasma bovis* are in demand. The objective of this longitudinal field study was to gain knowledge about the dynamics of antibodies against *M. bovis* in sera from naturally exposed calves with and without different clinical signs, measured by two different ELISA tests.

**Results:**

A total of 83 calves were subject to between one and five blood samples and clinical examinations using a standard protocol during five herd visits to each of four outbreak dairy herds. The blood samples were analysed for the presence of antibodies against *M. bovis* using the commercial IgG ELISA test BioX K302 (BioX) and an in-house indirect IgG ELISA test (MilA ELISA).

Linear mixed models were used to describe and compare the antibody dynamics as measured by the two tests in relation to the disease status and age of the animals.

The BioX ELISA response was below the recommended cut-off (37 ODC%) for the entire study period in many of the calves. The estimated mean ODC% increased slowly but did not reach the recommended individual animal cut-off in three of the four herds. The highest estimated ODC% was not reached until the calf was 110–130 days old. The MilA ELISA response rose above the recommended cut-off (135 antibody units (AU)) in almost all calves, and in two herds, the estimated mean was above the individual animal cut-off shortly after the birth of the calf. The highest estimated antibody concentration was reached when the calf was approximately 60 days old. Disease status of the calf was not significantly associated with the results of either test.

**Conclusions:**

We conclude that the BioX ELISA cannot be recommended for use in calves below 3 months of age. The MilA ELISA was able to detect antibodies shortly after birth (i.e. from approximately 3 weeks of age and onwards) and is therefore a more sensitive test for *M. bovis* exposure in young calves. Neither ELISA seemed able to differentiate between calves with arthritis and/or otitis media, and respiratory disease.

## Background

*Mycoplasma bovis* causes severe disease in cattle worldwide. The typical clinical manifestations in calves are pneumonia, otitis media and arthritis [1]. The primary diagnostic tool used in calves is bacterial culture of body fluid samples [2], but this is too expensive and time-consuming for use in group or herd diagnostics or for surveillance purposes. Although bacterial DNA-detection tests (such as PCR assays) are becoming more popular, the diagnostic material used for this technique is more difficult to obtain and process than a blood sample. An ELISA for antibody-detection is easy to perform on serum samples and is often less expensive, and these assays are already commonly used for the diagnosis of other diseases in cattle. Knowledge about the dynamics of antibody response in infected animals in relation to disease and age is essential when using an ELISA as a diagnostic tool. This knowledge requires longitudinal studies of naturally exposed calves, involving repeated observations of clinical signs combined with samples being taken for laboratory testing. However, this is time-consuming, inconvenient and expensive, and therefore rarely implemented, meaning that our existing knowledge about antibody dynamics in calves comes mostly from experimental studies. Calves vaccinated with an experimental aerosol vaccine against *M. bovis* at three to 4 weeks and five to 6 months of age appeared to have a detectable immunoglobulin G (IgG) response against *M. bovis* within 14 days, and the antibody concentrations in serum remained at a high level for at least 42 and 30 days, respectively [3].

However, neither the *M. bovis* antibody response to systemic disease syndromes, such as arthritis, or the dynamics of the antibody response over time in naturally infected calves is clear. It is also crucial to know how to interpret ELISA results in young calves, since maternally derived antibodies against *M. bovis* might be present in uninfected calves. Furthermore, very young calves may not be able to generate an antibody response to bacterial infections [4]. Other authors have found the antibody titres in young dairy calves to be low, suggesting low levels of passive transfer of antibodies from the dam [5, 6]. No correlation has yet been found between clinical signs and antibody response in individual calves, but seroconversion to *M. bovis* has been shown to be predictive of disease at a group level in feedlot cattle [7, 8]. To date, there have been few evaluations of the use and interpretation of different *M. bovis* ELISA tests under field conditions. A recent study found that the antibody response in cows was very dynamic, of short duration and dependent on clinical signs in the cow [9], but similar studies in calves under different herd and disease conditions are lacking.

An in-house IgG-detection ELISA (MilA ELISA) developed by Wawegama et al. [10] has an estimated animal-level sensitivity and specificity of 94.3% and 94.4%, respectively using a cut-off of 105 antibody units (AU) [8]. This study compared the results from the MilA IgG ELISA with those obtained from the BioX K302 and K260 ELISA assays in two small groups of experimentally infected calves, and found that both BioX tests had low sensitivity.

To the best of our knowledge, there has been no comparison of the antibody responses measured using the MilA ELISA and the BioX K302 ELISA (BioX) in dairy calves, and knowledge about the generation of antibodies in serum in naturally exposed calves with and without different clinical signs is lacking. Therefore, the objective of this longitudinal field study was to describe and compare the dynamics of antibody responses to *M. bovis* in the serum of dairy calves with different disease manifestations using two different ELISA tests.

## Methods

Data for this study were collected from four dairy herds, from which both cows and calves were sampled. A description and an analysis of the data from cows are presented in Petersen et al. [9], while analyses of the data from calves are presented here. The basic study design and herd selection were the same for the two studies. All farm owners were informed about the procedures in the study and gave written consent to use of their animals and farm data before study start. The study design was approved by the veterinary department of the agricultural advisory services, SEGES, before initiation.

### Study design

A longitudinal observational study was carried out between 1st July 2015 and 5th April 2016 in four Danish dairy cattle herds with acute outbreaks of *M. bovis-*associated disease. Each herd was visited five times with an interval of approximately 3 weeks between each visit. The first visit was as close as possible to the onset of the disease outbreak. The clinical status of selected calves was assessed at each visit, and blood samples were collected from them. Where possible, the same animals were sampled again at each subsequent visit, allowing both between- and within-animal analysis over time.

### Study population

The study herds were selected based on the detection of *M. bovis-*associated clinical signs by the herd advisory veterinarian and by diagnostic test results (positive in PCR assays on milk samples or in ELISA tests on sera from cows or calves). All herds had a recent history of sudden-onset of clinical signs indicative of *M. bovis* infection among the cows and/or calves, and several strongly positive ELISA and/or PCR test results for *M. bovis.*

All herds tested positive in both an ELISA and a PCR assay on at least one occasion during the study period, but the clinical signs present in the herds and the affected age groups differed. Information about the farms is presented in Table 1.

**Table 1:** Summary description of the four Danish dairy herds and diagnostic test results prior to and during the study period

Herd no.	1	2	3	4
Prior to enrolment				
Herd size (No. of cows)	177	174	182	391
Estimated start of outbreak	Early Jun 2015	Early Jul 2015	Late Nov 2015	Mid Dec 2015
After enrolment				
Data collection	1 Jul 2015–16 Sep 2016	20 Jul 2015–6 Oct 2015	8 Dec 2015–23 Feb 2016	20 Jan 2016–5 Apr 2016
Age group primarily affected	Cows	Cows and calves	Cows	Cows and calves
Primary clinical signs				
Cows	Mastitis	Mastitis, arthritis	Arthritis	Mastitis, arthritis
Calves	Pneumonia, otitis media	Arthritis, otitis media, pneumonia	Few cases of arthritis and otitis media	Arthritis, otitis media, pneumonia
Diagnostic tests				
Number of calves	15	22	20	26
Number of samples	51	101	89	93
Positive samples^a^				
BioX^b^	16	26	14	13
MilA^c^	48	98	41	88
Positive cultures from necropsied calves (total necropsied calves)	0 (0)	1 (1)	0 (2)	2 (2)

^a^Number of seropositive samples out of all sera obtained during the study period

^b^ODC% values > 37 in BioX Bio K 302 ELISA (BioX Diagnostics, Belgium)

^c^AU > 135 in the MilA ELISA

Laboratory analysis of calves that were euthanised and necropsied outside the planned project activities revealed additional information about the study herds. One four-month-old calf from Herd 2 had chronic degenerative arthrosis in several joints and bronchopneumonia with overlying pleuritis; *M. bovis* was cultured from joint fluid, and both joint fluid and lung tissue were PCR positive for *M. bovis*. Two one-month-old calves in Herd 4 had chronic omphalitis, bronchopneumonia, synovitis in several joints and bilateral otitis media; *Mycoplasma spp.* were cultured from these calves and identified as *M. bovis* by PCR*.*

Bacterial culture was negative for two seven-month-old calves from Herd 3 that were necropsied, even though arthritis and otitis media were observed in both animals. However, the presence of typical clinical signs (including arthritis/swelling of the limbs, and very high serum antibody titres against *M. bovis*) in multiple cows*,* and the failure to detect any other pathogens suggest that it is highly likely that the clinical signs were associated with *M. bovis*.

No animals from Herd 1 were necropsied, but positive PCR results from milk from cows with mastitis were obtained before and during the study period.

### Sample collection

The project budget allowed for approximately 400 tests in total, corresponding to five tests on each of 80 calves. However, during an outbreak of *M. bovis* some calves may die or be euthanized because of clinical disease, so we initially enrolled more than 80 calves in the study. During the first visit, the investigating veterinarian and the farmer sampled the calves strategically to ensure that a sufficient number of calves suspected to have *M. bovis-*associated disease were included, as well as a sample of calves without evidence of clinical disease. The rationale behind this non-random sampling strategy was to maximize the likelihood of including sufficient numbers of animals with and without different clinical signs.

During the herd visits, each calf underwent a clinical examination with a focus on the respiratory and musculoskeletal system, using a standardised clinical protocol, which is available from the corresponding author. One of three veterinarians performed the clinical examinations, and at least one of the authors of the clinical examination protocol was present at each visit.

In addition, a venous blood sample was collected from each calf using a 10 ml plain Vacutainer tube (Kruuse, Denmark). Blood samples were stored in a cool environment and delivered to the Eurofins Steins laboratory (Vejen, Denmark) within 36 h of collection. The serum samples were frozen at − 18 °C.

### Detection of antibodies

The serum samples were thawed at the National Veterinary Institute, in the Technical University of Denmark (DTU), Copenhagen, and analysed for antibodies against *M. bovis* using the commercial kit BioX Bio K 302 ELISA (BioX Diagnostics, Rochefort, Belgium) and the in-house indirect IgG ELISA test (MilA ELISA) developed at the University of Melbourne, Australia, by Wawegama et al. [10].

The BioX assay was performed according to the manufacturer’s instructions. The test outcome was calculated as:

ODC% = ((OD_sample_ – OD_negative control_) / (OD_positive control_ – OD_negative control_)) × 100.

Where OD is the optical density. An ODC% > 37 was considered positive, as recommended by the manufacturer. The manufacturer reported the sensitivity and specificity of the test at this cut-off to be 100% in a small sample of experimentally infected and negative control calves [11]. However, other authors have found that the assay has a low sensitivity in experimentally infected animals [12] and studies in cows under field conditions has supported this observation [9].

The MilA ELISA was performed as described by Wawegama et al. [10]. The mean antibody concentration in antibody units (AU) was calculated by plotting the OD values on a standard curve derived from a set of known positive-control sera included on each plate. In feedlot cattle with BRD the sensitivity and specificity of this assay have been estimated at 94.3% (95% confidence interval: 89.9–99.6%) and 94.4% (95% confidence interval: 90.3–99.6%), respectively, using 105 AU as cut-off [8]. However, the authors recommend using AU > 135 as cut-off for a test positive interpretation.

### Statistical analysis

Individual calves were categorised into the following disease groups:

1) Likely *M. bovis*-associated disease (‘*M. bovis*’).

2) Respiratory disease only (‘Respiratory’).

3) No clinical signs of disease (‘Healthy’).

This classification was based on the recorded clinical signs and photographs of each calf taken during the herd visits, using the following specific inclusion criteria:

- *M. bovis:* calves with clinical signs indicating infection with *M. bovis.* All calves in this group had arthritis and/or otitis media at one or more herd visits, and some of these calves also had signs of respiratory disease.
- Respiratory: calves with only clinical signs of respiratory disease that did not fall into the category given above. All calves had one or more of the following clinical signs at one or more herd visits: dyspnoea, abnormal lung sounds on auscultation, discharge from the nares or eyes and coughing.
- Healthy: calves with no clinical signs of disease.

### Modelling of antibody responses

Separate linear mixed models were used, with BioX and MilA ELISA results as the outcome variables. The ODC% and AU measurements were log transformed to improve the normality of the residuals, and a fixed constant of 1 was added to all results to enable log transformation of all values, including the small number of zero values.

For each of the two outcomes, model selection was used to find the most parsimonious model based on the potential explanatory variables and their two-way interactions. The fixed effects to be tested were: the disease group (categorical variable), the herd (categorical variable) and the age (in days) of the calf on the sampling date (pseudo-continuous variable). An additional quadratic effect of age was included in order to allow for a non-linear relationship between age and ELISA response. The final model was obtained using backward stepwise elimination based on Akaike’s information criterion (AIC). A random effect of calf identification number was included in all models in order to account for repeated samples from the same calf. Confidence intervals for the predicted mean ODC% and AU values (for an “average” calf) were calculated for each herd using parametric bootstrapping.

**Fig. 1:**
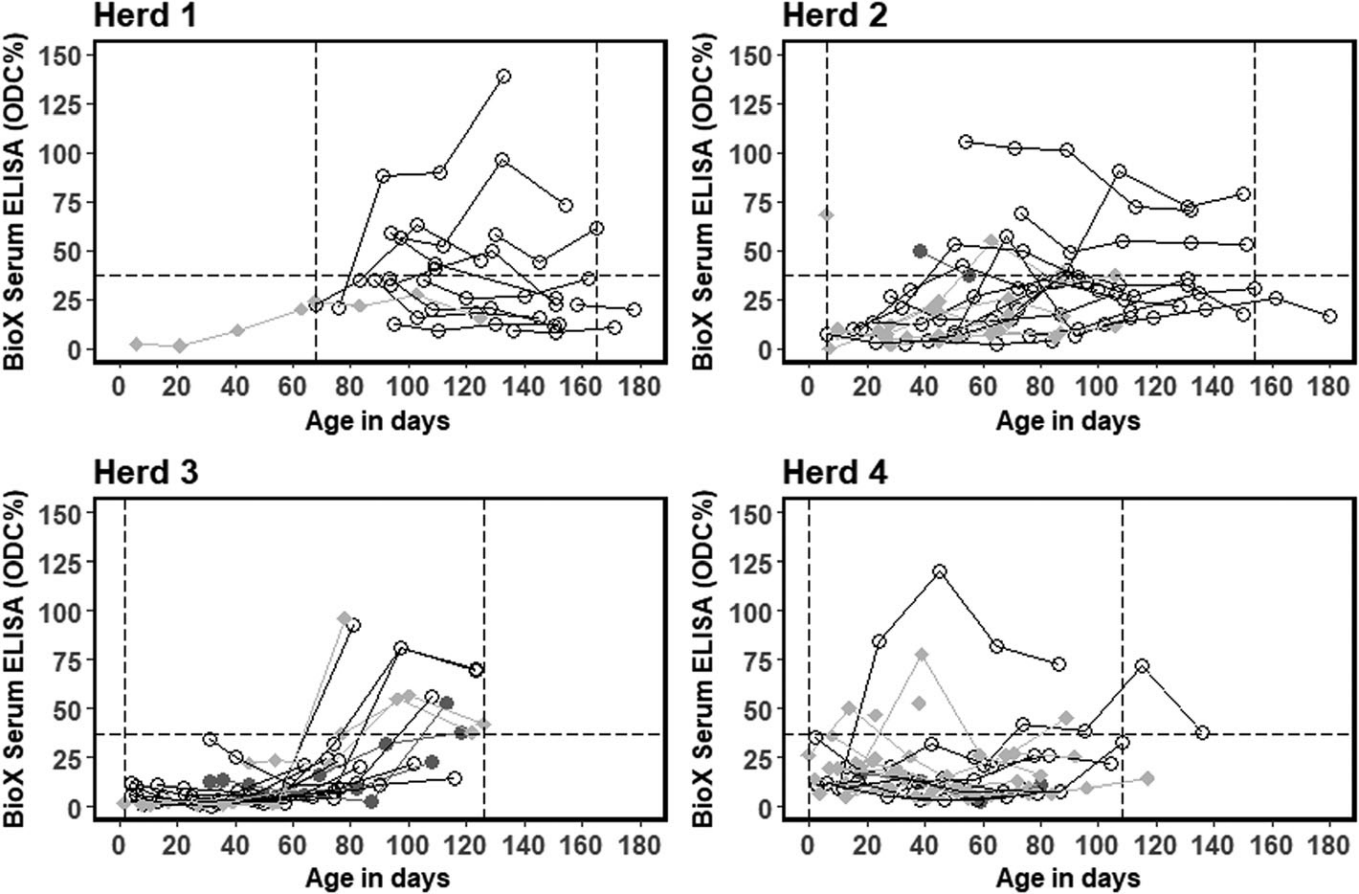
Distribution of ELISA measurements in the BioX ELISA Bio K302 assay (ODC% = sample coefficient) of serum antibodies against *M. bovis* in four Danish dairy herds. Grey squares = “*M. bovis”*; black circles = “Respiratory”; grey dots = “Healthy”. Horizontal dashed lines show the recommended ELISA cut-off (37 ODC%). Vertical dotted lines indicate the limits for including observations in the modelling of antibody response dynamics. Results from the same calf are linked by lines

**Fig. 2:**
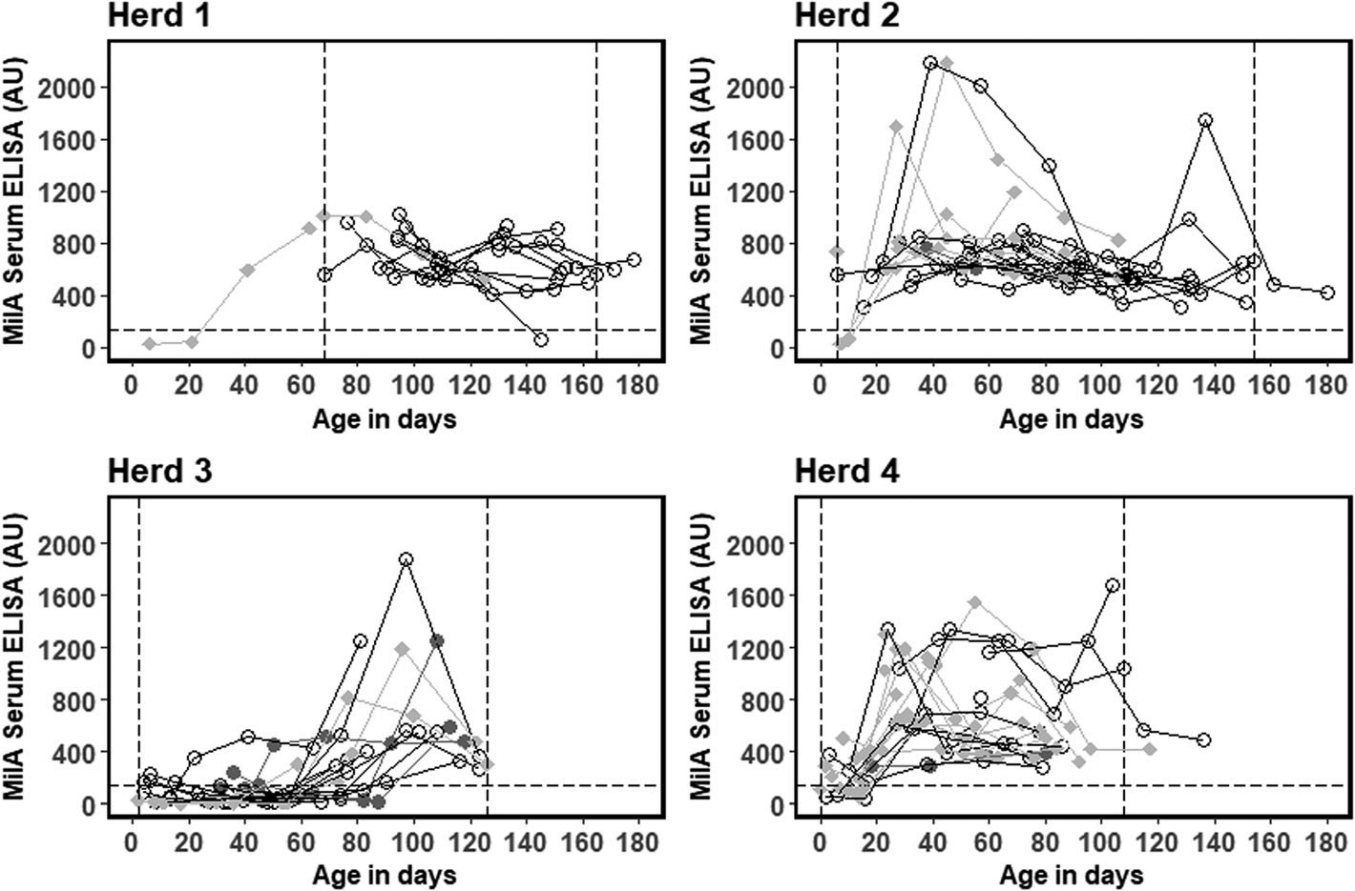
Distribution of serum ELISA measurements in the MilA assay (AU = sample antibody units) of serum antibodies against *M. bovis* in four Danish dairy herds. Grey squares = “*M. bovis”*; black circles = “Respiratory”; grey dots = “Healthy”. Horizontal dashed lines show the recommended ELISA cut-off (135 AU). Vertical dotted lines indicate the limits for including observations in the modelling of antibody response dynamics. Results from the same calf are linked by lines

Age intervals containing fewer than three observations from different calves in the same herd were removed. This was done because different age groups were sampled across the different herds, and to restrict the models to regions of parameter space with enough observations. As a result, herd-specific graphs of predicted ELISA responses span different age intervals for each herd. Dashed vertical lines in Figs. 1 and 2 illustrate the herd-specific age ranges included in the modelling.

The goodness of fit of the model was estimated using marginal and conditional pseudo *R*^*2*^ for mixed models, which was estimated using the method described by Nakagawa and Schielzeth [13]. Using this method, the marginal *R*^*2*^ describes the variation explained by the fixed effects alone, and the conditional *R*^*2*^ describes the variation explained by both fixed and random effects. All data management and analyses were done in R version 3.2.2 (R Core Team, 2016), with linear mixed models implemented using the lme4 package [14].

## Results

### Descriptive statistics

In total, 83 calves were enrolled in the study and 334 blood samples were collected. Table 2 shows the number of calves stratified by herd and disease group.

**Table 2:** Distribution of calves by disease group and herd in a Danish longitudinal field study of four dairy herds with an outbreak of *M. bovis*-associated disease

Disease group	*M. bovis*	Respiratory	Healthy	Total
Herd 1				
Calves	2	13	0	15
Samples	8	43	0	51
Herd 2				
Calves	7	14	1	22
Samples	29	70	2	101
Herd 3				
Calves	6	11	3	20
Samples	23	51	15	89
Herd 4				
Calves	15	10	1	26
Samples	49	40	4	93
Total				
Calves	30	48	5	83
Samples	109	204	21	334

Of the 83 calves, 52 were sampled on five occasions, 16 on four occasions, 1 on three occasions, 6 on two occasions and 8 on one occasion. The primary reason for calves dropping out of the study was euthanasia (*N* = 13), and eight calves were moved to another property during the study.

Due to a laboratory error, samples from the first visit to Herd 1 were not analysed.

The BioX ELISA response was below the recommended cut-off of 37 ODC% for the entire study period for 48 of the calves, above the cut-off for the entire study period for eight calves, and 27 changed status (Fig. 1). Very few calves had an ODC% above the cut-off before they were 40–60 days old (Fig. 1).

The MilA ELISA response was above the recommended cut-off of 135 AU throughout the entire study period for the majority of the calves (Fig. 2). Only one calf was below the cut-off at the end of the study period. The MilA ELISA detected antibodies soon after birth (i.e. at approximately 20 days of age), but as was evident in Herd 3, the antibodies did not increase quickly in all herds. The responses varied, but remained above the cut-off once it had been reached.

Based on the raw data plots, there seems to be no association between disease group and antibody responses for either ELISA test (Figs. 1 and 2), so disease group was not included as an explanatory variable in the statistical models.

### Modelling of antibody responses

**Fig. 3:**
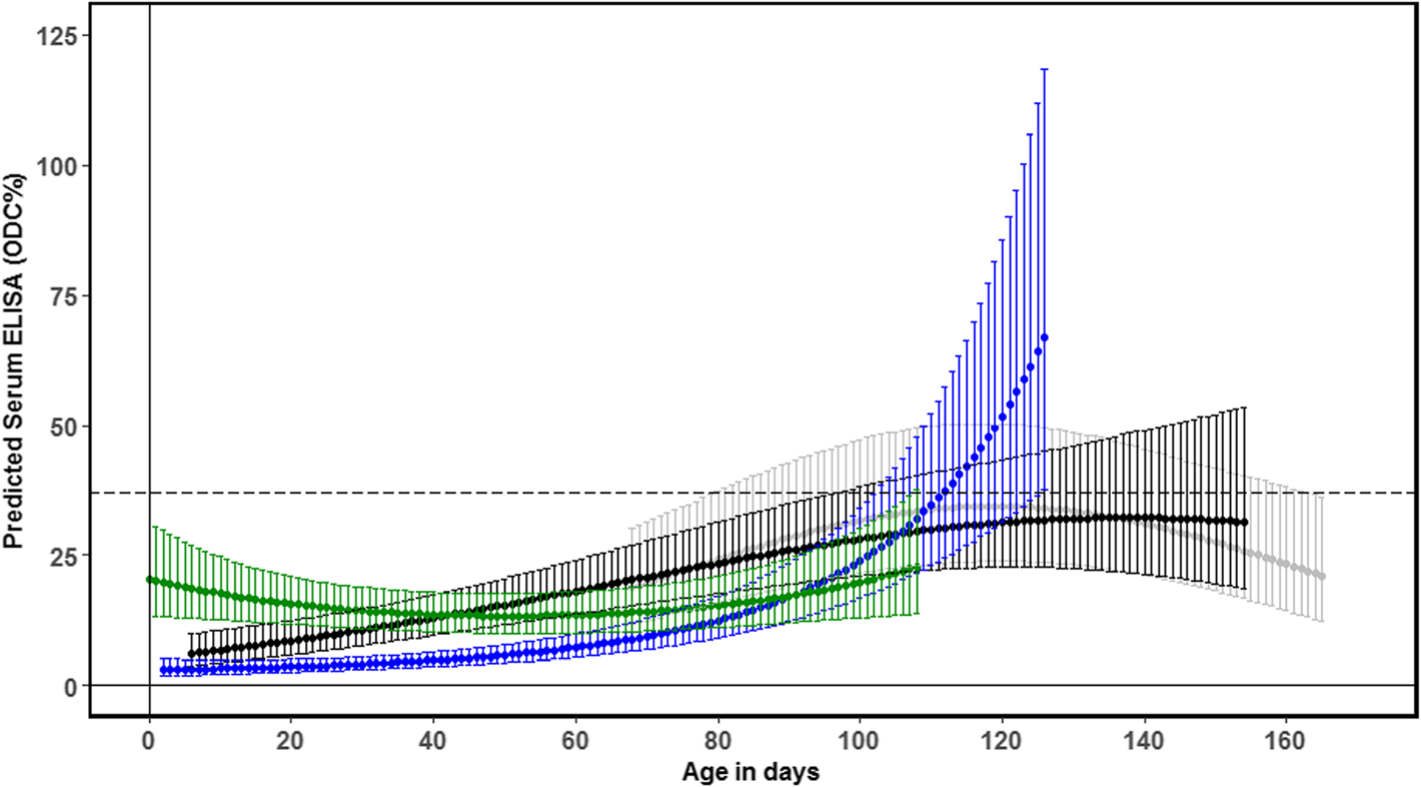
Estimated mean antibody response in sera (solid line) and 95% confidence intervals (shaded area) as measured by the BioX ELISA Bio K302 assay for the herd-specific age ranges for which observations were available. Herd 1 is grey, Herd 2 is black, Herd 3 is blue and Herd 4 is green. The dashed line shows the recommended individual animal ELISA cut-off (37 ODC%)

The final model with log transformed BioX ODC% as the outcome included the linear and quadratic effects of age, the fixed effect of herd, and the two-way interactions between herd and both the linear & quadratic effects of age (Table 3). The variance associated with the random effect of animal was considerable, although less so than the residual variance. There was a positive linear effect of age in all herds, but the sign of the quadratic effect was dependent on herd. Based on Fig. 3, the mean estimate of ODC% in three of the four herds increased gradually with age and did not reach the recommended individual animal cut-off. In the remaining herd, the rate of increase in ODC% increased with the age of the calf. The highest mean estimate of ODC% was not reached until the calf was approximately 110–130 days old (depending on the herd), with some suggestion of a plateau and eventual decline above this age in three of the herds (Fig. 3). However, a comparison of this relationship among herds is complicated by the difficulty in extrapolating the polynomial effect of age outside the observed parameter space, which was further compounded by the small differences in the ages of the calves among herds.

**Table 3:** Final model describing explanatory variables and random effects of log transformed BioX ELISA optical density measurements (ODC%). The marginal *R*^2^ was 39% and conditional *R*^2^ was 61%

Variables	Variance	95% confidence interval		
Random effects				
Animal	0.28	0.13–0.38		
Residuals	0.47	0.37–0.54		
		Estimate	SE	*p*-value
Fixed effects				
Intercept		2.55	0.26	< 0.001
Age days (linear)		14.68	3.93	< 0.001
Age days (quadratic)		−8.16	2.27	< 0.001
Herd 1		0		–
Herd 2		0.30	0.30	0.311
Herd 3		−0.10	0.30	0.753
Herd 4		0.37	0.31	0.229
Age days (linear)*Herd 1		0		–
Age days (linear)*Herd 2		−5.93	4.27	0.165
Age days (linear)*Herd 3		7.11	4.59	0.123
Age days (linear)*Herd 4		−8.15	5.06	0.108
Age days (quadratic)*Herd 1		0		–
Age days (quadratic)*Herd 2		4.80	2.65	0.072
Age days (quadratic)*Herd 3		13.42	3.19	< 0.001
Age days (quadratic)*Herd 4		13.95	3.31	< 0.001

**Fig. 4:**
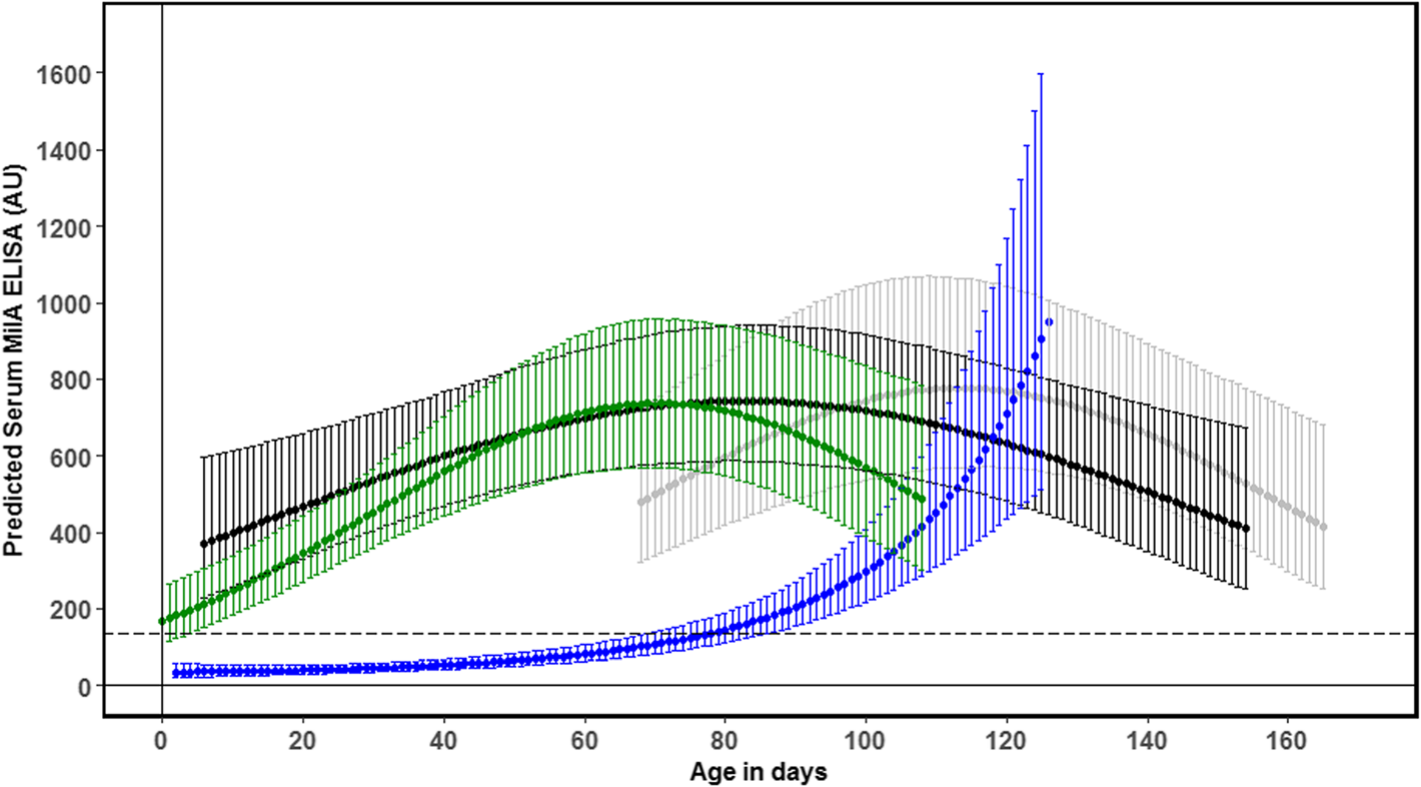
Estimated mean antibody response in serum (solid line) and 95% confidence intervals (shaded area) as measured by the in-house MilA ELISA for the herd-specific age ranges for which observations were available. Herd 1 is grey, Herd 2 is black, Herd 3 is blue and Herd 4 is green. The dashed line shows the recommended individual animal ELISA cut-off (135 AU)

The final model with log transformed MilA AU as the outcome also included the linear and quadratic effects of age, the fixed effect of herd, and the two-way interactions between herd and both the linear & quadratic effects of age (Table 4). The variance associated with the random effect of animal was less than that estimated for the BioX ELISA. Again, there was a positive linear effect of age in all herds, but as for the BioX ELISA, the sign of the quadratic effect was dependent on herd. Based on Fig. 4, it appears that the MilA ELISA detected antibodies in younger calves, and for two herds, the mean estimate of MilA AU was above the recommended animal cut-off value for animals less than 20 days old. The overall shape of the relationship between age and estimated AU was similar in three of the four herds (Fig. 4). It can be characterised by an initial phase of increase followed by a plateau and an eventual decrease, although the peak was reached at the older age of 110–120 days in Herd 1, compared to approximately 60–80 days in Herds 2 and 4. A significantly different pattern, which was more similar to an exponential increase from an initially low MilA AU value, was estimated for Herd 3 (Fig. 4).

**Table 4:** Final model describing explanatory variables and random effects of log transformed MilA ELISA antibody units (AU). The marginal *R*^2^ was 59% and conditional *R*^2^ was 65%

Variables	Variance	95% confidence interval		
Random effect				
Animal	0.09	0.007–0.150		
Residuals	0.57	0.463–0.655		
		Estimate	SE	*P*-value
Fixed effects				
Intercept		5.77	0.22	< 0.001
Age days (linear)		12.91	3.53	< 0.001
Age days (quadratic)		−8.25	2.18	< 0.001
Herd 1		0		–
Herd 2		0.61	0.25	0.015
Herd 3		−0.83	0.25	0.001
Herd 4		0.32	0.26	0.231
Age days (linear)*Herd 1		0		–
Age days (linear)*Herd 2		−11.90	3.89	0.003
Age days (linear)*Herd 3		10.79	4.35	0.014
Age days (linear)*Herd 4		−15.88	4.84	0.001
Age days (quadratic)*Herd 1		0		–
Age days (quadratic)*Herd 2		4.13	2.64	0.118
Age days (quadratic)*Herd 3		14.71	3.29	< 0.001
Age days (quadratic)*Herd 4		−2.19	3.40	0.520

## Discussion

This is the first observational study to illustrate and analyse the dynamics of serum antibody responses against *M. bovis* in naturally exposed and infected calves. In addition to a large variation in antibody responses among calves both within and between herds, the key findings were that the BioX ELISA rarely detected antibodies in calves under the age of 2 months, while the MilA ELISA was able to detect antibodies in the same calves soon after birth. Since the calves in these herds were all very likely to be truly exposed, we conclude that the MilA ELISA is a potentially useful test of *M. bovis* exposure. However, neither ELISA could differentiate between calves with arthritis and/or otitis, and respiratory disease, which indicates that the tests may be less useful for distinguishing animals with *M. bovis*-related diseases from those that have just been exposed to the pathogen.

### Antibody dynamics measured by the BioX ELISA

In general, very few calves seroconverted to values above the recommended cut-off of 37 ODC%, and the majority remained below the cut-off for the BioX ELISA for the entire study period, despite the fact that these calves either showed signs of *M. bovis-*associated disease or were housed with diseased calves during acute outbreaks of disease caused by *M. bovis*. In Herd 2, several calves had severe arthritis, and *M. bovis* was cultured from one necropsied calf. Despite this, the BioX ELISA did not detect antibodies above the recommended cut-off in sera from these calves (Fig. 1; grey triangles)*.* It is possible that because these calves were very young when they were infected, their immune system was not yet able to produce antibodies against the *M. bovis* antigen used in the BioX ELISA. A similar phenomenon has been seen with *Salmonella* Dublin [4] and Virtala et al. [5] also found that young calves often fail to seroconvert to common respiratory pathogens including *M. bovis*, although this was assessed using a different assay. The BioX ELISA was also used in a vaccine challenge study by Dudek et al. [15]. In the positive control group, which consisted of five- to six-week-old heifers intratracheally challenged with *M. bovis*, the antibody response increased only slightly to a maximum of approximately 50 ODC% at 4 weeks after challenge, and thereafter declined slightly. By contrast, in the vaccinated group, which was inoculated subcutaneously with inactivated *M. bovis* mixed with two adjuvants, serum antibody concentrations rapidly increased within 2 weeks of vaccination and reached a maximum of around 200 ODC% in 4 weeks. This difference is likely to reflect the greater stimulation of the systemic immune response following inoculation with an adjuvanted whole cell vaccine compared to natural infection.

Our study is the first to evaluate the dynamics of antibody responses as measured by the BioX ELISA in calves under field conditions and we have shown that it is not a suitable test for reliable diagnosis of *M. bovis* in calves under 3 months of age. However, this does not exclude the possibility that this test may be useful for group diagnostics in younger calves with an adjusted cut-off. Additional studies are needed to explore this possibility in herds with differing disease status.

### Antibody dynamics measured by MilA ELISA

The MilA ELISA detected antibodies above the cut-off in calves as young as approximately 20 days of age, with antibody concentrations rising markedly over a short timeframe, and at the end of the study period, all but one of the calves were above the cut-off. However, the MilA ELISA did not detect antibodies early in all four herds. In Herd 3, the antibody levels did not start to rise until the calves were 60–80 days of age, and then increased rapidly. No *M. bovis-*associated disease was found among the calves in this herd during the first two herd visits, indicating that they had managed to prevent transmission between cows and calves and had kept the infection pressure low around the calves. In addition, the first visit to Herd 3 was only 1 week after the appearance of clinical signs in the herd, and the farmer did not feed any waste milk to the calves. The later response detected by the MilA ELISA in this herd could be a result of initial infection among the cows and later transmission to the calves (i.e. around 40–60 days after the outbreak had started). The other herds were visited three to 4 weeks after the outbreak had started, and transmission to the calves had already occurred by this time. This could explain the high serum antibody concentrations in young calves in these herds. It can therefore be concluded that the MilA assay does not detect young calves as positive in non-infected groups of calves, which supports a reasonable specificity and therefore the usefulness of this test for confirmation and surveillance purposes. To substantiate this finding, control herds with no known *M. bovis-*associated diseases should be assessed for comparison. The high antibody concentrations detected in most calves in this study, including the healthy calves, suggests that the MilA test is very sensitive and probably detects exposure to *M. bovis* rather than *M. bovis*-associated disease. Both experimental and field studies of the MilA ELISA also suggest that this test has a high level of sensitivity [8].

### The use of antibodies to detect infection with *M. bovis*

Our study has shown that the *M. bovis* BioX and MilA ELISAs give very different results for calves with the same exposure and disease status in calves younger than 2 months old indicating different immune reactions to the underlying infection (Herd 2 and 4 in Figs. 1 and 2). In herds with clinical signs in older calves, the modelled antibody responses were similar in shape, indicating similar immune reactions to the underlying infection (Herd 1 and 3 in Figs. 1 and 2). This is likely associated with the age-related development of immune competence in the calves, which is important for the interpretation of the test results for diagnosis.

In addition to the disease burdens in the calves studied here, severe clinical signs associated with infection with *M. bovis* where found among the cows in all four herds, which is highly likely to have resulted in transmission to the calves in some of the herds. Fewer calves had severe signs of *M. bovis*-associated disease in Herds 1 and 3 than in Herds 2 and 4, i.e. herds with little or no segregation of cows and calves and feeding of unpasteurised whole milk to the calves. As *M. bovis* spreads by direct contact between animals and through ingestion of milk contaminated with *M. bovis* [1], this is likely to have contributed to the severity of the disease in these two herds. In Herds 1 and 3 the calves were fed milk replacer and housed in a building that physically separated them from the cows.

Clinical disease did not seem to correlate with the antibody response detected by either ELISA assay. The effect of “Herd” was retained during model selection, indicating that the different ELISA responses were mostly influenced by differences among the herds, and not differences in the underlying disease status of individual animals. Although Martin et al. [7] performed their study using an indirect haemagglutination assay, they also concluded that serum antibodies against *M. bovis* were not indicative of disease at an individual level, only at group level.

Only five calves were classified as being healthy, which is a small number to include in the models as a separate group, yet disease group was not found to be significant. To assess the robustness of the results, models were generated both without the ‘Healthy’ group, and with the healthy calves included in the ‘Respiratory’ group, and none of these variations altered the conclusions.

The ‘Respiratory’ group consisted of many calves, and it is not possible to know whether the disease in this group was caused by *M. bovis* alone or in conjunction with other respiratory pathogens*.* At the time of sampling, the calves were housed in herds with an active or recent spread of *M. bovis,* so it is likely that the disease seen was at least partly attributable to infection with *M. bovis*. In Herds 2 and 4, *M. bovis* was isolated from necropsied calves, and all herds were free of the likely differential diagnosis *Salmonella* Dublin throughout the study period, making it likely that the arthritis was caused by *M. bovis.*

## Conclusions

This is the first study to evaluate the dynamics of antibody responses using the BioX ELISA in calves under field conditions. Based on our data, we cannot recommend the use of this test in calves under 3 months of age. The MilA ELISA was able to detect antibodies shortly after birth (i.e. from approximately 3 weeks of age and onwards) and is likely to be a good assay for detecting exposure to *M. bovis*. Neither ELISA could differentiate between calves with arthritis and/or otitis media, and respiratory disease.

## Abbreviations

AU: antibody units
BioX: BioX Bio K 302 ELISA test
ELISA: enzyme-linked immunosorbent assay
IgG: immunoglobulin G
*M. bovis*: *Mycoplasma bovis*
MilA: in-house indirect IgG ELISA test
OD: optic density
ODC%: corrected optic density measurement
PCR: polymerase chain reaction
